# A national survey of services for the prevention and management of falls in the UK

**DOI:** 10.1186/1472-6963-8-233

**Published:** 2008-11-12

**Authors:** Sarah E Lamb, Joanne D Fisher, Simon Gates, Rachel Potter, Matthew W Cooke, Yvonne H Carter

**Affiliations:** 1 Warwick Clinical Trials Unit, Warwick Medical School, University of Warwick, Coventry CV4 7AL, UK; 2 Kadoorie Critical Care Research Centre, John Radcliffe Hospital, Oxford, OX3 9DU, UK; 3 Warwick Medical School, University of Warwick, Coventry CV4 7AL, UK

## Abstract

**Background:**

The National Health Service (NHS) was tasked in 2001 with developing service provision to prevent falls in older people. We carried out a national survey to provide a description of health and social care funded UK fallers services, and to benchmark progress against current practice guidelines.

**Methods:**

Cascade approach to sampling, followed by telephone survey with senior member of the fall service. Characteristics of the service were assessed using an internationally agreed taxonomy. Reported service provision was compared against benchmarks set by the National Institute for Health and Clinical Excellence (NICE).

**Results:**

We identified 303 clinics across the UK. 231 (76%) were willing to participate. The majority of services were based in acute or community hospitals, with only a few in primary care or emergency departments. Access to services was, in the majority of cases, by health professional referral. Most services undertook a multi-factorial assessment. The content and quality of these assessments varied substantially. Services varied extensively in the way that interventions were delivered, and particular concern is raised about interventions for vision, home hazard modification, medication review and bone health.

**Conclusion:**

The most common type of service provision was a multi-factorial assessment and intervention. There were a wide range of service models, but for a substantial number of services, delivery appears to fall below recommended NICE guidance.

## Background

The prevention of falls in older people is an increasingly important focus of health policy in many industrialised societies that are experiencing an ageing population. Approximately 30% of people aged over 65 fall each year, and this proportion rises to 80% for those aged 80 years and older (reviewed in [1] and [2]). For some, the consequence of a fall can include serious injury and increased dependency [1,2]. In the UK, the government has responded to this agenda through a number of targeted policy initiatives. The first initiative was the National Service Framework (NSF) for Older People which was published in 2001 and required the English National Health Service (NHS) to establish fall-prevention programmes[1]. Little operational guidance was provided until a review and clinical guideline undertaken by the NHS policy body, the National Institute for Health and Clinical Excellence (NICE), was published in 2004[2]. NICE undertakes a formal and well structured approach to guideline development, based on systematic reviews, expert appraisal of evidence, and integration of evidence, expert and user opinion. The NICE fall guideline mandated that older people should be screened for risk of falling in an opportunistic manner when presenting in primary care, secondary care, and other settings. Further assessment and intervention is indicated for those individuals who report a fall in the last year and have a gait or balance problem. NICE identified the essential elements of a falls assessment to be gait/balance; osteoporosis risk, medication review, home hazard and vision, based on the strength of the evidence base in 2004. NICE concluded that individualised multi-factorial interventions should include interventions on strength and balance training, home-hazard intervention, modification or withdrawal of medications, and referral for correction of visual defects as appropriate. A number of other interventions were reviewed (e.g. cardiac interventions), but owing to lack of evidence, guidance relating to these interventions was less definitive. NICE set out a series of standards for the UK NHS (based on the above) and in addition recommended that programmes should be flexible enough to accommodate participants' different needs and preferences, and should promote the social value of such programmes. Finally, NICE suggested specialist falls services should be operationally linked to bone health (osteoporosis) services.

The most cost effective method of providing falls services is not known, and in 2004, NICE launched an economic appraisal of different approaches [2]. This was subsequently suspended because of lack of information regarding existing services. The aim of the survey reported here was to map the organisational structure, service provision and processes of falls services funded by health and social services in the UK to inform this economic modelling project. One focus of the analysis was to compare the provision of assessment and intervention in five key areas (gait/balance, vision, medication, home hazards and bone health (i.e. osteoporosis services)) to the benchmarks set by the NICE guidance[2]. We aimed also to elicit information on a broader range of services.

## Methods

### Sampling

We aimed to include all fallers' services within each health region (in the UK these are defined as Primary Care Trust (England, Wales and Northern Ireland) or health board (Scotland)). We wrote to the lead clinician/manager/director of all Primary Care Trusts (PCTs), Physiotherapy Departments, Geriatric (or equivalent) Departments, Emergency Departments (ED) and Social Services in the UK, requesting contact details for services. This meant that multiple letters were sent to each health organisation in the UK. In addition, we identified ED-based fallers services from the Department of Health/British Association of Emergency Medicine funded survey of emergency department services in the UK [3], and posted requests for information on the interactive Chartered Society of Physiotherapy website and our own project website. A reminder letter was sent to non-responders after six weeks.

We conducted a standardised telephone interview with the lead clinician/manager/director (or their designate) of each service. Characteristics of the clinic were summarised using the taxonomy of fall prevention interventions developed with collaboration from the Prevention of Falls Network Europe[4]. The taxonomy was developed by international expert consensus as a tool to assess the main components of fall prevention interventions, and develop definitions of service components by either utilising existing international classification systems, or developing additional classifications where these were not already available. The taxonomy includes assessment of the primary aims of the service, the selection criteria used (demographic, chronic diseases, symptoms or impairments, medication specific), the environment from which participants were identified and where services were delivered, details on individuals providing assessment and interventions (self-assessment/management, professionals, trained non-professionals, institutions, others), the design of the intervention (details on assessments provided), types of intervention (single, multiple or multi-factorial), and finally, descriptions of the main types of intervention provided (supervised exercise (individual/group), type of exercise, medication withdrawal or modification, surgery, urinary incontinence, fluid or nutrition therapy, psychological interventions, environmental/assistive technologies, social environment interventions, knowledge (including advice)). Full details including the definitions of all terms are available at . In addition, we collected data on the organisations overseeing the services' activities, the relationship to other service providers, whether interventions were provided by the service, or by onward referral to other services, referral routes, and relationships to other local amenities and services. NHS Trust reports were used to estimate the base population of services, including the ethnic and age mix of the local population; the socio-demographic index, and whether the service was based in a rural or urban or mixed area.

### Ethical approval

Research Ethics Committee approval was not required as the project fell under service evaluation (confirmed by National Research Ethics Service, June 2006).

### Analysis

We detailed service profiles across acute and community settings, using frequency analysis. Chi-squared(χ^2^) tests were used to investigate associations between service provision and setting. Missing data were checked rigorously by re-telephoning clinics. If the respondent did not know whether a clinic provided a particular service, or was unable to find out from colleagues, the clinic was coded as not providing that service. As missing data were on average less than 5% our results are not likely to be significantly biased in this respect. The data were analysed using the SPSS statistical software package (version 14, SPSS Inc).

## Results

**Figure 1:**
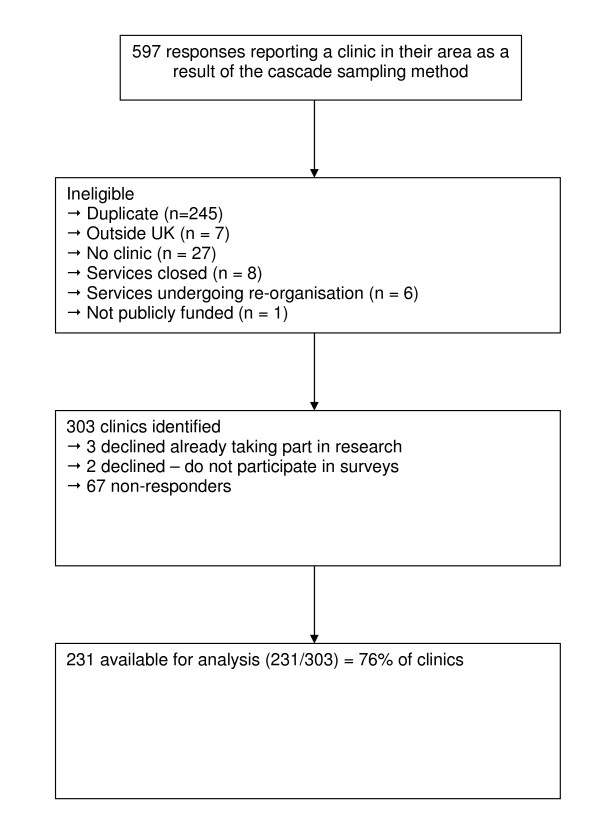
Flow chart of survey response.

Details on response, uptake and reasons for declining to participate in the survey are shown in Figure 1. A total of 2744 request letters were mailed between 1^st ^June and 31^st ^July 2006. Once duplicate reports and ineligible responses were removed, 303 separate services were identified, covering all UK health regions. Of these, 67 services did not respond to the invitation for a telephone interview and 5 declined to participate. The final sample size was 231 services (76% of all services identified).

**Figure 2:**
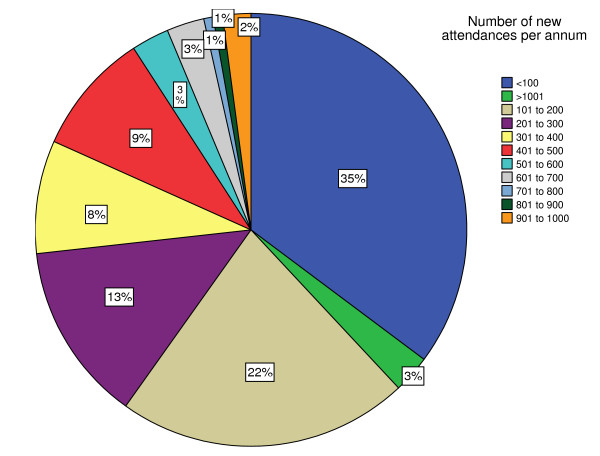
**The percentage of clinics by number of new attendances per annum. **Total number of clinics providing data on number of new attendances per year was 142.

Service characteristics are detailed in Table 1. The most common base was a hospital (222/231, 96%), with a near-equal division between community and acute hospitals. The most usual method of entry into a service was referral from a health or social care professional (143/231, 62%). In addition, a minority of services accepted referrals from self, family, nursing homes, voluntary agencies, care and/or home support services (78/231, 34%). Most services used falls, near falls or fear of falling to determine eligibility. A minority of services used a screening tool with published evidence of validity (51/231, 22%), the most common being the Falls Risk Assessment Tool [5] (29/231, 13%). The predominant staffing structure was a multi-disciplinary team (212/231, 92%), although less than 30% of services had the combination of a physiotherapist, nurse, occupational therapist and doctor (full multi-disciplinary team). Acute sector hospitals were more likely to include a doctor (73% versus 41%) and to have a full multi-disciplinary team (40% versus 22%). Otherwise there were no major differences between acute and community services. The median number of attendances per year was 180 (Figure 2, based on 142 services).

**Table 1:** Characteristics of services (denominator is 231 unless otherwise stated)

		**Number (%) N = 231**
Location	Urban	142 (61%)
	Rural	48 (21%)
	Mixed	41 (18%)

Base	Primary care	2 (1%)
	Emergency department	5 (2%)
	Intermediate care hospital	5 (2%)
	Community (social services or other)	7 (3%)
	Acute hospital (excluding E.D.)	105 (45%)
	Community hospital	107 (46%)

Referral	Health/social care professional	143 (62%)
	Self-referral	78 (34%)
	Doctor only	8 (3%)
	Other	5 (2%)
	Missing	2 (1%)

Eligibility Criteria (not mutually exclusive)	None	18 (8%)
	Falls, near falls or fear of falling	170 (74%)
	Screening tool (own unpublished)	89 (39%)
	Age	82 (35%)
	Use of 3 or more medications	58 (25%)
	Screening tool (published)	52 (23%)

Age	Over 60 years only	145 (63%)
	All people over 15	82 (35%)
	Missing	4 (2%)

Staffing structure	Multi-disciplinary (MDT)	212 (92%)
	Single discipline	18 (8%)
	Missing	1 (< 1%)
	MDT included physiotherapist†	187 (88%)
	MDT included nurse†	163 (77%)
	MDT included OT†	162 (76%)
	MDT included doctor†	123 (58%)
	MDT included physiotherapist, nurse, OT and doctor†	70 (33%)

† Denominator = 212 multidisciplinary teams

Table 2 summarises the reported provision of multi-factorial assessment and intervention. Nearly all services undertook multi-factorial assessments (228/231, 99%). The methods and components of the assessment varied substantially. The majority of services assessed gait and balance, home hazards and/or medication (> 72%). Overall, 25 different gait and balance assessment methods were reported, ranging from self-report to timed and observed performance tests. Cardiovascular assessments were reported by 69%. The majority of clinics used simple assessments including auscultation, self-report and bed-side postural hypotension tests. Vision assessments were provided by 58% of clinics, but the majority was by self-report. A Snellen chart or formal vision assessment was used by 13% of clinics (29/231). Less than half of the services reported undertaking any assessment of bone health/osteoporosis (107/231, 46%).

**Table 2:** Details of assessments and interventions provided by services (denominator is 231 unless otherwise stated)

		Number (%) N = 231
Multi-factorial risk assessment	Not undertaken	3 (1%)
	Undertaken	228 (99%)
	Includes gait and balance	210 (91%)
	Includes home hazards	176 (76%)
	Includes medication	167 (72%)
	Includes cardiovascular	160 (69%)
	Includes vision	135 (58%)
	Includes cognition	124 (54%)
	Includes foot	123 (53%)
	Includes nutrition	118 (51%)
	Includes bone health	107 (46%)
	Includes hearing	80 (35%)

Multi-factorial intervention	Reported using interventions	192 (83%)
	linked to a multi-factorial	
	assessment	

Knowledge/information intervention	No information provided	13 (6%)
	Written information	215 (93%)
	Video information	26 (11%)
	Audio information	22 (10%)
	Formal education program	112 (48%)

Gait and balance intervention	Exercise supervised in clinic	182 (79%)
	Home exercises	104 (45%)
	Referral to community class	48 (21%)

Medication intervention	Intervened (any type)	99 (43%)
	Direct	61 (26%)
	Onward referral	38 (16%)

Bone health Intervention	Intervened (any type)	55 (24%)
	Direct	30 (13%)
	Onward referral	25 (11%)

Vision Intervention	Intervened (any type)	81 (35%)
	Direct action	8 (3%)
	Onward referral	73 (32%)

Cardiovascular Intervention	Intervened (any type)	38 (16%)
	Direct action	9 (4%)
	Onward referral	29 (13%)

Home hazard Intervention	Intervened (any type)	138 (60%)
	Direct action	80 (35%)
	Onward referral	59 (26%)

Incontinence Intervention	Intervened (any type)	96 (42%)
	Direct action	15 (6%)
	Onward referral	81 (35%)

Foot health intervention	Intervened (any type)	68 (29%)
	Podiatry	20 (9%)
	Onward referral	48 (21%)

Hearing intervention	Intervention (any type)	45 (19%)
	Direct action (ear wax removal)	37 (16%)
	Onward referral	8 (3%)

Post-intervention follow-up	Face to face	75 (32%)
	Post	1 (< 1%)
	Telephone	28 (12%)
	Combination	9 (4%)

The components of the multi-factorial interventions varied between services, the most common combination being knowledge provision, exercise and medication intervention. Environmental, vision and bone health interventions were less frequent. There was a notable discrepancy between the number of services providing assessment (228/231, 99%) and those following on with a linked multi-factorial intervention (192/231, 83%). The remainder of services provided knowledge or exercise only. For all services, knowledge was usually provided in a written format (93%), with a few clinics (3/231) using audio or video information only. Some clinics supplemented their information provision with a formal educational program (112/231, 48%), most commonly educational talks (109/112), but 6% of clinics reported providing no information. Exercise was used by 81% of clinics (188/231). The majority of exercise programmes were undertaken at the service location (182/231, 79%), with the remainder using a home or a community based programme. The most usual form of exercise was strength, gait and balance training. The mean duration of the exercise programmes was 8 weeks (SD 2.96; range 2–24), and the mean number of sessions per week was 1 (SD 0.04). Home hazard modification (including grab rails, raised seats, lighting, trolleys and removal of rugs) was provided by 60% of services. Less than 30% of services dealt with aids for personal mobility, signalling devices, hip protectors and/or safe footwear. Interventions for vision were reported by 35% (81/231) of the services, predominantly by onward referral. Overall, 43% of services dealt with medication issues, with 26% of services reporting taking direct action to modify or discontinue medications and 16% (38/231) referring patients to the GP, pharmacist or consultant for prescription modification. Even fewer intervened on bone health; 13% of services reported prescribing calcium, vitamin D and/or bisphosphanates, and 11% (25/231) referred to GP, consultant or pharmacist for this. Post-intervention follow-up was undertaken by 113/231 services (49%). The length of the follow-up period varied from 2 to 52 weeks (mean 21 weeks, SD 16.9).

## Discussion

The main finding of this survey is that multi-factorial assessment and intervention is the most common form of NHS falls service. Services have been established, but there is now a substantial concern that significant numbers of services are failing to attain the standards set for multi-factorial programmes by NICE. There is substantial variability in content and quality of screening, assessment and interventions currently provided, and a failure by many services to implement procedures that are supported by research evidence.

Falls services developed rapidly in the UK after the National Service Framework for Older People in 2001[1]. The NHS was advised to develop falls services as a matter of priority, although very little practical guidance was available. In 2004, NICE provided more detailed guidance[2], and set out recommendations for the core elements of services. This guidance was based on a systematic review of the evidence base. NICE recommended that all individuals who were at risk of falling should receive written information, and the majority of services have achieved this. Compared to the total population of people over the age of 65 living in the UK (11 million), and the expectation that at least a third of these individuals will fall each year[6], reports of attendance suggest that the population reach of fall services is low (< 3% of the population at risk) [7].

Information/knowledge provision is the most common component of services. Didactic educational programmes are in common use, despite several randomised trials suggesting this to be an ineffective method of promoting behavioural modification, risk and fall reduction [8,9]. Further research should develop effective written materials given their predominance in falls management [10]. Gait/balance assessments and exercise interventions are provided by many services. In comparison to interventions of known effectiveness [11,12], the distribution of the number of sessions per week, and the duration of programmes, suggests that at least some services maybe utilising sub-optimal levels of exercise. Further research is needed to determine the dose-response relationship of exercise and reduced falls.

We ascertained whether services dealt with specific target risk factors by direct action (providing a treatment), or by onward referral. Even considering both of these, intervention on home hazards, medication, vision and bone health was low in comparison to the NICE recommendations. Some services provided assessment of these risk factors, but did not provide either a treatment or onward referral to deal with the risk amelioration other than information provision. Recent systematic reviews point toward potential inadequacies of multi-factorial interventions that rely on information and/or onward referral [13], and raise the possibility that single interventions maybe as effective as multiple and multi-factorial interventions in some populations [14]. Further research is needed to determine effective and cost-effective service delivery models.

It is important to consider whether methods used in the survey may have biased the findings. We elicited the full range of services provided from a senior clinical or managerial lead. This is a different method than used in the National Clinical Audits of Falls and Bone Health (NCAFBH) [15], which traced the journey of patients who had sustained a hip or wrist fracture to determine the services received. With the organisational survey approach it is unclear whether all components of the service are utilised appropriately, and the results may suffer from reporting bias. The survey method does capture information on a much broader range of services aimed at the entire population of people at risk of falling. However, despite different methods, the NCAFBH and our organisational survey have come to similar conclusions, that services are established, the population reach is low, and that the quality of delivery is disappointing in key areas of bone health, medication review, and vision assessment. Comparison against the NCAFBH[15] and National UK Survey of Emergency Services[3] supports our confidence that we have identified the substantial majority of services. The next question is whether or not there is a difference between services who participated in the survey and those who did not. With a response rate of 76% of potentially eligible services, we have captured the majority of services. We were unable to collect extensive data from services that did not participate, but available information suggests that they were not significantly different from those participating.

In conclusion there is a need to improve falls service provision[16]. The quality of assessments and interventions need to be improved and coverage needs to be increased. Given current uncertainties in the evidence base[13,14], we suggest that future refinements to fall services should be underpinned by randomised evaluations to determine effectiveness and cost-effectiveness of competing service delivery models.

## Conclusion

There is a need to improve the quality and reach of falls service provision in the UK.

## Abbreviations

ED: Emergency Department; GP: General Practitioner; NCAFBH: National Clinical Audits of Falls and Bone Health; NHS: National Health Service; NICE: National Institute of Health and Clinical Excellence; NSF: National Service Framework; PCT: Primary Care Trust

## Competing interests

The authors declare that they have no competing interests.

## Authors' contributions

SL, SG, MC and YC were responsible for obtaining the funding and for the concept of the original study. SL and SG oversaw the design, conduct and analysis of the survey. JF and RP refined the survey design, undertook data collection, and analysis. All authors were involved in the interpretation of the data. The final manuscript was prepared by SL, and approved by all authors.

## Pre-publication history

The pre-publication history for this paper can be accessed here:


